# Surrogate Decision‐Making Regarding Life‐Sustaining Treatments for Older Adults with Dementia Admitted to Long‐Term Care Hospitals

**DOI:** 10.1002/alz.090085

**Published:** 2025-01-09

**Authors:** Hyejin Kim, Jeonghyun Cho, Jongsun Park, Sang Suk Kim

**Affiliations:** ^1^ Chung‐Ang University, Seoul, Seoul Korea, Republic of (South); ^2^ Inje University, Busan, Busan Korea, Republic of (South)

## Abstract

**Background:**

Long‐term care hospitals are the main healthcare setting for the growing population with dementia in South Korea. Despite various deficiencies (e.g., insufficient resources, impractical policies and regulations) in providing quality end‐of‐life care in this care setting, few studies have explored decision‐making practices in the context of dementia and end‐of‐life care planning. Thus, this study aimed to explore how decisions concerning life‐sustaining treatments were made for patients with dementia admitted to long‐term care hospitals, identify facilitators and challenges in the process, and suggest directions to improve the current decision‐making practices.

**Method:**

This qualitative descriptive study included 24 healthcare providers—physicians, registered nurses, social workers, or oriental medicine doctors involved in dementia care for at least six months in their current long‐term care hospitals—and 7 surrogates—family members involved in medical decision‐making. We conducted individual, semi‐structured interviews with the participants regarding decision‐making approaches, facilitators and challenges to decision‐making, and suggestions to improve decision‐making practices. Data were analyzed using a directed content analytic technique.

**Result:**

The analysis yielded 2 main categories, 7 categories, and 19 subcategories: *How decisions about life‐sustaining treatments were made for patients with dementia?*; and *How to support decision‐making about life‐sustaining treatments for this patient population?* Physicians, nurses, and surrogates were the key personnel in decision‐making about life‐sustaining treatments in long‐term care hospitals and played different but complementary roles. Although their primary goal of care was to maintain patients’ current functional status as long as possible while securing comfort, they showed three different views toward life‐sustaining treatments. When making decisions, they considered patients’ autonomy and best interests, surrogates’ interests, and other ethical factors. Various patient/surrogate/staff‐related factors facilitated or challenged decision‐making about life‐sustaining treatments. Individual/institution/policy/regulation‐level efforts were suggested to support such decision‐making.

**Conclusion:**

The findings highlight the importance of open communication between healthcare providers and surrogates regarding end‐of‐life care for patients with dementia. Multi‐faceted strategies (e.g., the promotion of advance care planning, education to enhance healthcare providers’ competency in end‐of‐life care discussions, development of policies and regulations supporting life‐sustaining treatment‐related practices in long‐term care hospitals) are necessary to effectively assist surrogates in treatment decision‐making in this care setting.

